# Prospects and Challenges in Developing mRNA Vaccines for Infectious Diseases and Oncogenic Viruses

**DOI:** 10.3390/medsci12020028

**Published:** 2024-05-22

**Authors:** Lakshmi Venkata Simhachalam Kutikuppala, Islam Kourampi, Ramya S. D. Kanagala, Priyadarshini Bhattacharjee, Sri Harsha Boppana

**Affiliations:** 1 Department of General Surgery, Dr. YSR University of Health Sciences, Vijayawada 520008, India; 2 Department of Medicine, National and Kapodistrian University of Athens, 11527 Athens, Greece; 3 Department of Medicine, Dr. KNR University of Health Sciences, Warangal 506007, India; ramyasdkanagala@gmail.com; 4 Cambridge University Hospitals NHS Foundation Trust, Hills Road, Cambridge CB2 0QQ, UK; p.bhattacharjee1@nhs.net; 5 Department of Anesthesia and Critical Care Medicine, Johns Hopkins School of Medicine, Baltimore, MD 21205, USA; sboppan2@jh.edu

**Keywords:** infectious diseases, mRNA technology, vaccine research, virus induced cancers

## Abstract

mRNA vaccines have emerged as an optimistic technological platform for vaccine innovation in this new scientific era. mRNA vaccines have dramatically altered the domain of vaccinology by offering a versatile and rapid approach to combating infectious diseases and virus-induced cancers. Clinical trials have demonstrated efficacy rates of 94–95% in preventing COVID-19, and mRNA vaccines have been increasingly recognized as a powerful vaccine platform. Although mRNA vaccines have played an essential role in the COVID-19 pandemic, they still have several limitations; their instability and degradation affect their storage, delivery, and over-all efficiency. mRNA is typically enclosed in a transport mechanism to facilitate its entry into the target cell because it is an unstable and negatively charged molecule. For instance, mRNA that is given using lipid-nanoparticle-based vaccine delivery systems (LNPs) solely enters cells through endocytosis, establishing an endosome without damaging the cell membrane. The COVID-19 pandemic has accelerated the development of mRNA vaccine platforms used to treat and prevent several infectious diseases. This technology has the potential to change the future course of the disease by providing a safe and effective way to combat infectious diseases and cancer. A single-stranded genetic sequence found in mRNA vaccines instructs host cells to produce proteins inside ribosomes to elicit immunological responses and prepare the immune system to fight infections or cancer cells. The potential applications of mRNA vaccine technology are vast and can lead to the development of a preferred vaccine pattern. As a result, a new generation of vaccinations has gradually gained popularity and access to the general population. To adapt the design of an antigen, and even combine sequences from different variations in response to new changes in the viral genome, mRNA vaccines may be used. Current mRNA vaccines provide adequate safety and protection, but the duration of that protection can only be determined if further clinical research is conducted.

## 1. Introduction

Despite advancements in conventional vaccine procedures, issues persist, prompting the creation of innovative vaccine technologies. Viral infections cause epidemic outbreaks that recur nearly every year. These outbreaks always come on suddenly, are extremely morbid, spread quickly, and have negative social impact [1]. A “vaccine on demand” model that enables speedy vaccine development, mass production, and delivery would be ideal. Current vaccination technology platforms, which usually require time-consuming and labor-intensive research and development operations, would not be compatible with such an approach [2]. Due to their power to elicit broadly protective immune responses and their ability to be synthesized via quick and adaptive production techniques, nucleic-acid-based vaccines, such as viral vectors and mRNA, can be suited for rapid response applications [3]. The production of vaccines can be completed more quickly and at a lower cost by using the same manufacturing facilities, production, and purification processes for all vaccines built on the same nucleic-acid platform. This is conceivable because nucleic-acid-based vaccines are producible without using encoded antigens [4]. Because nucleic-acid-based vaccines mimic a viral infection to produce vaccine antigens in situ, they result in cytotoxic T cell and humoral responses after immunization. The elimination of intracellular pathogens or infections, which demands potent humoral and cellular immune responses to be successful, is dependent on this advantage. Compared with immunizations using viral vectors, mRNA-based vaccines have various benefits [1,5]. Complex antigens can be expressible by mRNA vaccines that are not limited by packing specifications and that can provide for in situ antigen production without getting through the nuclear membrane barrier for protein expression. They also do not alter the host cell genomes or produce contagious particles. It is possible to create mRNA vaccines quickly, maybe within days of learning the gene sequence, utilizing entirely synthetic production techniques. The versatility and suitability of the mRNA platform for a wide range of targets make it ideal for speedy responses to newly identified diseases [2,6].

## 2. Evolution of mRNA Technology for Vaccine Research for Infectious Diseases and Virus-Induced Cancers (Figure 1)

Years of study and preparation have led to the successful production of mRNA vaccines. Brenner and colleagues first described the mRNA molecule in 1961, but, because of the mRNA molecule’s extremely fragile nature, it was not until 1969 that the first protein was generated in vitro from isolated mRNA [1]. The concept of mRNA vaccines was first proposed by Wolff et al. (1990), and they have been under development for the last 30 years [7].

**Figure 1:**
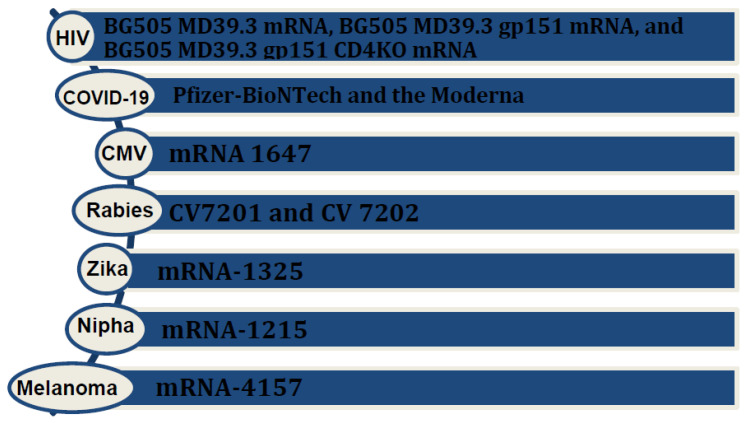
Various mRNA vaccines available or under investigation for infectious diseases and cancers.

The first “RNA interference (siRNA)” to be used as a therapeutic medicine—Onpattro^®^ (patisiran) (Alnylam Pharmaceuticals Inc., Cambridge, MA, USA) —was approved by the U.S. Food and Medicine Administration (FDA) in August 2018 after years of research [2]. The fundamental tenet of the technology underlying mRNA vaccine is based on a carrier system that enables nucleic acid delivery encoding the targeted antigen into the target cell in the human host, enabling the host cell to produce the targeted protein and express the targeted antigen to elicit an immune response. In reaction to the invasion of a pathogen carrying the antigen, the host’s immune system quickly initiates humoral and cellular immunological responses, finally putting an end to the illness [3]. Due to its negatively charged and inherently unstable nature, mRNA is often encapsulated in a delivery vehicle before reaching the target cell. Specifically, when utilizing lipid-nanoparticle-based vaccine delivery systems (LNPs), mRNA enters cells exclusively through endocytosis. This process ensures the establishment of an endosome without causing damage to the cell membrane. The endosome, after entering the cytoplasm, is sent directly to lysosomes for destruction [4]. Therefore, endosomal fusion with the lysosomes, and resulting disruption, must be avoided to guarantee structural stability and, consequently, translation of injected mRNA. According to studies [5], ionizable lipids in LNPs are involved in mRNA release and endosomal escape. In the endosomal acidic environment, the headgroup of the ionizable lipid undergoes protonation, turning it into a cation. The main benefit of mRNA vaccines is that mRNA undergoes translation by ribosomes into proteins, that are then used as endogenous antigens, and broken down into antigenic peptides by the proteasome. These peptides are then delivered to CD8+ cytotoxic T lymphocytes through the MHC class I molecular pathway to initiate cell-mediated immunity [6].

### 2.1. mRNA Vaccines in Infectious Diseases

Research on mRNA vaccines for HIV, particularly focusing on an HIV-1 Gag mRNA vaccine employing polyethyleneimine stearic acid (PSA), has yielded promising results. One study demonstrated notable potential, and elicited a positive immune response [8]. Lately, NIH launched a phased trial, known as the HVTN 302 study, for three mRNA HIV vaccines; BG505 MD39.3 mRNA, BG505 MD39.3 gp151 mRNA, and BG505 MD39.3 gp151 CD4KO mRNA. These vaccines are prepared to present spike protein, located on the surface of HIV, that facilitates its entry into human cells. Each of them encodes for different but highly related proteins [9]. Results of the HIV nanoparticle vaccine known as eOD-GT8 60-mer showed a successful production and expansion of a special type of B immune cell in nearly all the recipients. The team is currently collaborating with Moderna to produce the mRNA version of the eOD-GT8 60-mer [10].

In the context of the Zika virus, Moderna’s mRNA vaccines, namely mRNA-1325 and mRNA-1893, have recently concluded their human phase 1 clinical trials. The results indicate that these vaccines are well-tolerated and generate a robust immune response. These promising outcomes provide encouragement for advancing to further clinical trials [11].

Morbidity from cytomegalovirus (CMV) in transplant patients and immunocompromised groups made it necessary to make an efficient vaccine. The mRNA vaccine, mRNA 1647, designed to target cytomegalovirus (CMV), consists of six mRNAs. Among these, five encode the CMV pentamer complex, while one encodes the glycoprotein B (gB) protein. Notably, both antigens—the CMV pentamer complex and gB protein—are highly immunogenic. This composition underscores the vaccine’s potential efficacy, as demonstrated by its ability to trigger a strong immune response. The results of the latest study in humans showed durable and better antibody responses to the mRNA 1647 vaccine [12,13].

The CV7201 rabies virus mRNA formulation was the first to enter clinical trials in 2017. CV7201 and CV 7202 completed phase 1 clinical trials and demonstrated efficient efficacy and immunogenicity [14].

The first human clinical trial for a cancer therapeutic vaccine, incorporating PSA RNA transfected dendritic cells, represents a pivotal step in advancing innovative approaches to cancer treatment [15]. So far, more than 20 mRNA-based vaccines have been tested in clinical trials against solid tumors, including melanoma, non-small-cell lung cancer, and colorectal carcinoma. In the majority of these trials, mRNA cancer vaccines are given in conjunction with checkpoint modulators (PD-1, CTLA-4, and TIM3) or mixtures of cytokines to boost their effectiveness against tumors [16].

### 2.2. mRNA Vaccine in Melanoma

Exploring innovative approaches to melanoma immunotherapy, the mRNA vaccine Melanoma FixVac (BNT111) stands out as a liposomal RNA (RNA-LPX) vaccine, administered intravenously. It targets four non-mutated, tumor-associated antigens that are common in melanoma. This vaccine is currently being tested in an ongoing phase I trial, known as the Lipo-MERIT trial, on patients with advanced melanoma either alone or combined with the blockade of the checkpoint inhibitor PD1. It has produced objective responses in patients [17].

### 2.3. Personalized mRNA Vaccine

**Figure 2:**
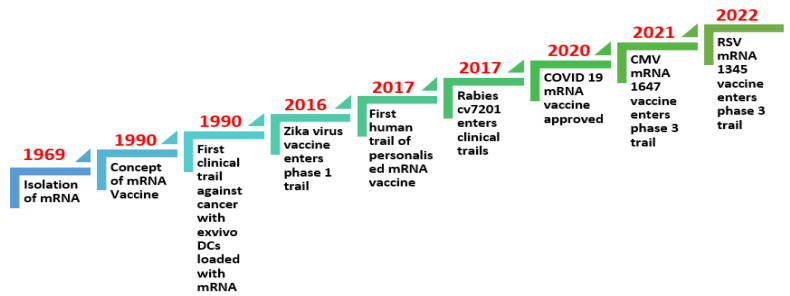
Timeline of mRNA technology evolution for vaccine research for infectious diseases and virus-induced cancers [21,22,23].

A significant milestone in the evolution of mRNA vaccines is the development of personalized vaccines. For instance, mRNA 4157, a personalized cancer vaccine that can encode up to 34 patient-specific tumor neoantigens, showed promising results in the KEYNOTE 942 trial. This trial showed an almost 45% reduction in risk of recurrence or death in patients who received mRNA 4157 in combination with pembrolizumab, compared with those who only received pembrolizumab [18]. In the context of another phase 1 clinical trial, the personalized neoantigen vaccine, autogenous cevumeran, employing uridine mRNA–lipoplex nanoparticles, demonstrated safety and viability when administered alongside atezolizumab and mFOLFIRINOX. It produced a significant number of neoantigen-specific T cells in half of the unselected patients with operable pancreatic ductal adenocarcinoma. This study is currently in phase 2 clinical trials [19,20] (Figure 2).

## 3. Vector Optimization for Efficient Delivery of mRNA Vaccine

During the outbreak of coronavirus disease 2019 (COVID-19), mRNA vaccines and replication-incompetent adenovirus (Ad) vectors were used widely for the first time. The humoral and cellular immunogenicity of mRNA vaccines has been evaluated in a holistic approach compared with the adenovirus vector industry, where antibody and cellular responses have traditionally been investigated separately [24]. Although mRNA vaccines have played a crucial role in the COVID-19 pandemic, they still have several limitations: their instability and degradation affect their storage, delivery, and overall efficiency. According to the literature, it has been proposed that, to achieve improved protein expression, variations in functional half-time and optimization of the codons are of high importance [25].

For the effective delivery of mRNA vaccines, numerous non-viral techniques have been proposed. Cationic nano emulsions (CNEs), polyplexes, and, specifically, lipid nanoparticles (LNPs) are used in these delivery techniques [26,27,28].

Lipid nanoparticles have undergone extensive research and successfully made their way into healthcare settings for the delivery of small molecules, siRNA drugs, and mRNA.

Notably, two authorized COVID-19 vaccines, Moderna (mRNA-1273) and Pfizer (BNT162b), utilized lipid nanotechnology to deliver antigen mRNA. Lipid nanoparticle–mRNA compositions demand several external and intracellular changes to operate in vivo. Secondly, after systemic administration, the formulation needs to evade capture by the MPS and renal clearance. Target tissues must be reached in the third step before internalization by target cells can occur. mRNA molecules must escape endosomes and enter the cytoplasm for translation to happen [29]. By altering the mRNA’s structural components like the 5′ cap, 5′ and 3′ UTR coding regions and the polyadenylation tail, one can constantly increase the stability and translational effectiveness of the mRNA and lessen its excessive immunogenicity [30].

The second-generation vaccines are produced after fixing the flaws of first-generation mRNA vaccines and improving their safety, effectiveness, storage, and handling while keeping the same efficacy and safety. The adjustments entail making the vaccines stable at room temperature and lowering the need for a cold chain for storage and transit. Finding more powerful and ligand-targeted nanocarriers with improved safety and mRNA-delivery-effectiveness profiles is another challenge. Additionally, there is a lot of ongoing study into the potential of using self-amplifying RNA and other RNA-based compounds such as vaccines [31].

The typical lipid thin film hydration procedure has been utilized to make liposomes and LNP. The formation of nanoparticles with different particle sizes has several disadvantages, one of which is the heterogeneous particle size distribution. To create consistent, homogenous LNPs, a later size-tuning strategy is now required [32].

mRNA is a negatively charged and large substance; various techniques were developed for its delivery into cells. These can be classified broadly as viral or non-viral vector delivery systems. Non-viral is further classified into lipid and polymer-based delivery systems. Lipid delivery systems can be liposomal complexes or lipo-nanoparticle delivery systems [33].

## 4. Recent Advances in mRNA Vaccines

The development of mRNA vaccine technology has brought immense hope to the world in mitigating and preventing diseases. Recent breakthroughs in the field of mRNA vaccines have allowed for the rapid advancement of vaccines for COVID-19 and have provided evidence for the viability of this novel vaccine modality [34,35]. The COVID-19 pandemic has been an ultimate test for mRNA-based vaccines and they have exceeded expectations, leading to the FDA’s authorization of the world’s first COVID-19 vaccine. This innovative technique has the potential to revolutionize how current medicine approaches protein-replacement therapy, cancer immunotherapy, immunization, etc. mRNA vaccines are a remarkable weapon for fending against infectious illnesses and impending pandemics [36,37,38,39,40,41,42].

The success of mRNA vaccines in preventing severe illness and hospitalization due to COVID-19 has been remarkable and has led to the authorization of Pfizer (BNT162b2) and Moderna (mRNA-1273) vaccines for market use in COVID-19 prevention [43,44,45,46,47,48,49]. mRNA vaccines have also shown effectiveness against new variants of COVID-19, emphasizing the promising potential of this technology in preventing infectious diseases [50,51]. The first COVID-19 vaccine to receive FDA approval for marketing and use in children aged 5 to 11 was the Pfizer-BioNTech vaccine. To date, 17 mRNA vaccines are under clinical research for COVID-19 prevention. The speed and ease of mRNA vaccine production make it a promising technology for responding to fresh changes in the viral genome, such as those that have emerged during the COVID-19 pandemic [52,53,54,55,56].

These breakthroughs are based on at least three decades of scientific development [57]. Critically significant improvements in mRNA vaccine development have been made during the past few years, including the creation of extremely effective and secure mRNA vaccine delivery technologies [37]. The quick and low-cost mass manufacture of next-generation mRNA vaccines will also be made possible by novel production techniques and delivery mediums. Another innovation is the creation of techniques for the quick, easy, and mass manufacturing of mRNA under cGMP guidelines, which will enable the production of uniformly high-quality vaccines [38]. Innovations in engineering mRNA sequences have also allowed for the modification of mRNA 5′ and 3′ UTRs, resulting in increased translational activity [39,40]. Combining a new imidazole-modified lipid, DOG-IM4, with conventional helper lipids can increase the thermostability of mRNA/LNP [41]. The immunostimulatory activity of mRNA can be altered chemically by changing the nucleotides of the RNA [42]. These breakthroughs have allowed mRNA-based vaccines to reach the market of mass vaccination for the first time at a relatively high speed, as demonstrated by Comirnaty^®^ (Pfizer, New York, NY, USA/BioNTech, Mainz, Germany) and Spikevax^®^ (Moderna, Cambridge, MA, USA) [43,44]. Additionally, the production of therapeutic and preventative vaccinations based on mRNA has promise for the treatment of infectious diseases. One of the most promising technologies for worldwide immunization, controlling infectious diseases, and creating new treatments is RNA-based products [45,46,47].

Unlike traditional vaccines, mRNA vaccines are non-infectious and are easily degraded. They do not integrate into the host genome and are well-tolerated with no serious health effects associated with their use, making it possible for broader use of mRNA vaccines in the future. The mRNA technology platform will make it possible to cure additional diseases and prevent and manage infectious diseases [52,53]. The success of mRNA vaccines in the COVID-19 vaccine race has drawn attention to their potential application in cancer therapy and protein-replacement therapies [54].

By inducing self-immune responses, they can improve our toolbox for treating established and reemerging communicable illnesses and malignancies. Clinical use has shown potential for the modularization of mRNA vaccine design and manufacture addressing various application situations [58]. While mRNA-based vaccines have shown promising results in terms of safety and efficacy, further testing is necessary to prove safety and efficacy in human beings at large [59]. In the following five years, crucial clinical trials of mRNA vaccines, particularly those against COVID-19, will be finished, giving researchers a deeper understanding of the platform for mRNA vaccines and their many delivery mechanisms. The development of mRNA vaccines can improve our capacity to respond to, and manage, newly developing communicable illnesses [60]. mRNA-based vaccines have significant advantages over traditional vaccines in terms of efficacy, safety, economic production, and large-scale production, making them a promising alternative to conventional vaccines [61]. The technology has the potential to change the future course of disease by providing a safe and effective way to combat infectious diseases and cancer [62,63]. A single-stranded genetic sequence found in mRNA vaccines instructs host cells to produce proteins inside ribosomes to elicit immunological responses and prepare the immune system to fight infections or cancer cells [64]. The potential applications of mRNA vaccine technology are vast and can lead to the development of a preferred vaccine pattern [65].

MRNA history goes back to 1990, when Wolff et al. demonstrated the synthesis of a target protein following intramuscular (IM) injection in mice [66]. Nonetheless, it took many years for the clinical validation of this new technology, primarily due to challenges related to its unstable nature and delivery [67]. At present, over 190 companies and research organizations are actively involved in advancing more than 310 mRNA vaccines and treatments. The development stages of these medications span from initial discovery and preclinical investigations to diverse phases of clinical trials. Globally, 125 of these products are currently in the clinical pipeline, with vaccines constituting two-thirds and therapeutics making up the remaining one-third. It is noteworthy that, excluding mRNA COVID-19 vaccines, most of these products are still undergoing early clinical testing [68].

A novel method for addressing pancreatic cancer is advancing to broader accessibility for patients in the next phase. Following findings from a preliminary study, a phase II clinical trial has been initiated to assess the efficacy of employing an mRNA vaccine in combatting one of the most lethal forms of cancer. This recent trial aims to determine if the therapeutic vaccine can lower the likelihood of pancreatic cancer recurrence post-surgical tumor removal. Approximately 260 patients will be enrolled in the study and the results from the phase 1 trial indicate that mRNA vaccines are not only safe but also have the potential to induce a sustained immune response. Among the 16 patients examined, 8 showed the activation of robust immune T cells in response to the vaccines. Those individuals who demonstrated a vigorous immune response experienced extended intervals before cancer recurrence, contrasting with patients who did not exhibit an immune response to the vaccine [69]. The mRNA-4157 vaccine encodes up to 34 patient-specific neoantigens and, based on genomic sequencing, takes about six weeks to manufacture. The companies plan to explore the therapy’s effectiveness in non-small-cell lung cancer. The success contrasts with historical challenges in anti-cancer vaccines, showcasing the potential of mRNA vaccines in targeting multiple neoantigens [70]. Melanoma’s unique responsiveness to immunotherapy remains a puzzle, possibly linked to the quality rather than quantity of neoantigens. Despite previous setbacks, the mRNA vaccine approach offers promise, leveraging immunotherapy success and personalized targeting for some cancers. Clinical trials are currently in progress to evaluate CRISPR-modified primary human T cells as a groundbreaking treatment for metastatic gastrointestinal cancer [71]. This innovative therapy aims to target an intracellular checkpoint, cytokine-inducible SH2-containing protein (CISH), previously deemed undruggable. Importantly, it was designed to maintain cell viability and function.

Randomized, placebo-controlled trials in pursuit of a safe and effective Zika virus vaccine involved healthy adults aged 18 to 49, assessing safety, immunogenicity, and Zika-virus-specific neutralizing antibodies (nAbs). mRNA-1325, tested in the USA, demonstrated general tolerability across dose levels (10, 25, and 100 μg) but elicited modest nAb responses. mRNA-1893 vaccine, tested in the USA and Puerto Rico, exhibited mostly mild to moderate adverse reactions at higher doses, yet induced robust and persistent Zika-virus-specific nAb responses in all participants by day 57, irrespective of their flavivirus serostatus. These favorable results, supporting the development of mRNA-1893 against Zika virus, highlight its tolerability and ability to generate strong nAb responses [72].

Another target in the sights of mRNA vaccine development is the Nipah virus. This zoonotic virus primarily spreads through animals, but person-to-person transmission can occur, leading to severe outcomes like coma or death. With no licensed vaccine or treatment currently available for Nipah virus infection, the National Institutes of Health (NIH) has initiated an early-stage clinical trial to evaluate an investigational vaccine targeting prevention. Beyond infectious diseases, extensive clinical trials are underway to explore mRNA vaccines’ efficacy against various cancers [69,73,74]. Additionally, a clinical trial has been ongoing focused on utilizing the adenine base editor (ABE) to treat sickle cell disease [75] (Table 1).

## 5. Improving the Stability of mRNA Vaccines for Infectious Diseases and Virus-Induced Cancers

mRNA vaccines have revolutionized the field of vaccinology by offering a versatile and rapid approach to combating infectious diseases and virus-induced cancers. However, their inherent instability poses a significant challenge to their widespread application. In recent years, researchers have dedicated their efforts to improving the stability of mRNA vaccines, aiming to enhance their efficacy, storage, and distribution [50,51,52]. The stability of mRNA vaccines is a critical factor in their efficacy and successful implementation. Overcoming the challenges associated with mRNA instability has been a primary focus of recent research efforts. Various strategies have been observed to enhance the stability of mRNA vaccines, offering promising solutions for their broader utilization in fighting infectious diseases and virus-induced cancers [53,54].

One key approach is the improvement of lipid nanoparticle (LNP) formulations used for mRNA delivery. LNPs have the potential to protect mRNA molecules and enhance their stability. Zhang et al. discussed the engineering of LNPs to optimize mRNA encapsulation, stability, and cellular uptake, thus improving vaccine efficacy [53]. Similarly, Allen and Mout explored the use of stable lipid formulations for the efficient delivery of mRNA vaccines [54]. Modified nucleosides have emerged as another avenue for stabilizing mRNA. Feng et al. (2023) focused on the rational design of modified nucleosides, which can enhance mRNA stability and protect the mRNA molecule from degradation, contributing to increased vaccine durability [55].

Moreover, the optimization of mRNA sequences and modifications plays a vital role in enhancing stability. Li and Wang (2022) highlighted strategies such as codon optimization and the incorporation of modified nucleotides to improve mRNA stability and translation efficiency, leading to more effective protein synthesis [41]. Temperature stability is a significant concern for mRNA vaccines, particularly during storage and transportation. Chen and Kim (2022) discussed approaches to enhancing the thermostability of mRNA vaccines by incorporating stabilizing agents and modifying RNA structures. These strategies aimed to protect the mRNA molecule from degradation under different temperature conditions, ensuring vaccine efficacy and extending shelf life [56]. Formulation strategies are being developed to address stability challenges in mRNA vaccines. Riedmann and Cooney (2022) discussed various formulation approaches, including the use of stabilizing excipients and protective coatings, to overcome the inherent instability of RNA therapeutics. These strategies aimed to preserve mRNA integrity during storage and delivery [57]. The stability and delivery challenges associated with mRNA vaccines were comprehensively reviewed by Lallana and Rincón-López (2022). They provided an overview of the strategies employed to enhance stability, as well as the complexities involved in their delivery, shedding light on the potential solutions for overcoming these hurdles [58].

Overall, the continuous advancements in stabilizing mRNA vaccines offer promising prospects for improving their efficacy and practicality [59,60]. Through the refinement of LNP formulations, rational design of modified nucleosides, optimization of mRNA sequences, and formulation strategies, researchers are paving the way for more stable mRNA vaccines. These advancements not only enhance the storage and distribution potential of mRNA vaccines but also contribute to their widespread utilization in combating infectious diseases and virus-induced cancers [61,62].

## 6. Formulation and Delivery of mRNA Vaccines

**Figure 3:**
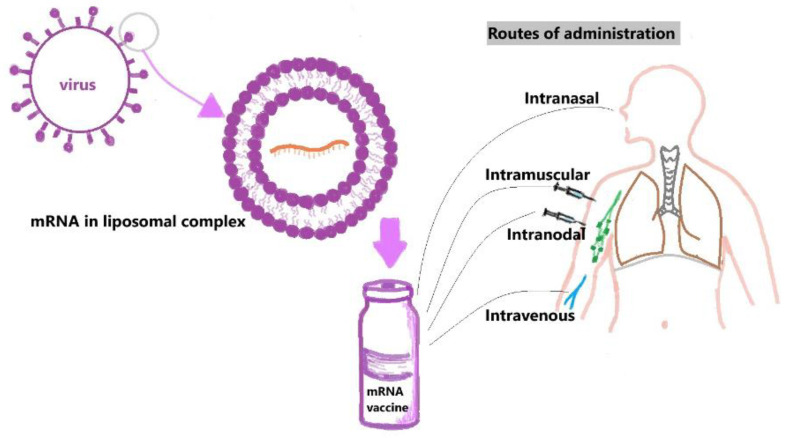
Diagrammatic representation of liposomal-complex vaccine development and common routes of administration of mRNA vaccines.

The steps of formulation and delivery of mRNA vaccines are as follows:

- Injection of naked mRNA:

Naked mRNA can be delivered directly after reconstitution with an appropriate buffer-like Ringer lactate or Ringer’s solution [63,64]. However, they are easily susceptible to RNases and have a limited ability to cross the lipid bilayer, which can be overcome by administering the drug locally by various methods like intramuscular, intra-nodal, and intra-nasal routes to minimize the vaccine contact with RNases in the bloodstream. Naked mRNAs are being tested currently in clinical trials for melanoma and hepatocellular carcinoma [65,66] (Figure 3).

b. Liposomal complexes:

Liposomes consist of single or multiple layers of phospholipids; they are positively charged cationic lipids with a core consisting of mRNA vaccine and, hence, they are not accessible to RNase. However, these liposomal complexes are positively charged, even in physiological conditions, which makes them easily susceptible to degradation [67,68].

c. Lipid Nanoparticles:

LNPs are composed of auxiliary lipids, a lipid bilayer shell, polyethene glycol, cholesterol, and an aqueous core in which the mRNA vaccine is present. LNPs comprise a mixture of various lipids that confer their physical stability, unlike the liposomal complexes [69,70].

d. Modification of LNPs:

Lack of target specificity, short blood-circulating time, and instability in vivo are some of the limitations of LNPs which can be overcome by modification. This modification includes targeting the liposomes with surface-bound ligands. For example, the folate receptor and transferrin receptor are overexpressed in cancer cells and have been targeted with liposomes using their corresponding ligands. Folate receptors have strong binding to folic acid, allowing for specificity in targeting tumor cells. Similarly, EGFR is overexpressed on many cells in cancers like non-small-cell lung cancer, and colorectal and breast cancer, where it can be used as a target to direct the drug to cancer cells [73].

e. Stimulus-responsive liposomes:

Drugs are released from modified stimulus-responsive liposomes in response to triggers such as changes in pH, temperature, enzymes, light, magnetic and electrical fields, and ultrasound. Given that the body has several pH gradients, pH change is the most promising trigger. When triggered, liposomes undergo a phase transition, allowing increased membrane permeability and a burst release of the drug. Temperature-responsive systems have undergone thorough investigation for delivering anti-cancer drugs, where local hyperthermia triggers lipids to approach their liquid–crystalline phase transition temperatures, resulting in drug release within tumors. Doxorubicin, for example, is temperature- and pH-sensitive [74,75].

f. Polymer-based delivery systems:

Polymer-based mRNA delivery systems offer specific capabilities, including the ability to form nanostructures in aqueous environments, undergo lyophilization, and exhibit distinct pharmacokinetics. However, their limited transfection efficacy and possible toxic effects pose challenges. Explicit polymer engineering, controlling chemical structure, and ensuring high reproducibility are essential for their translation into therapeutics [76,77].

Other nanoparticle materials include ferritin nanoparticles. Ferro nanoparticle vaccine has been shown to eliminate the hepatitis B virus in mice, and many clinical trials are ongoing [78,79].

## 7. Limitations of mRNA Technology

From the lab to the clinical setting, mRNA-based delivery systems face numerous challenges due to their extraordinarily large size, intrinsic instability, charge, and increased vulnerability to enzyme degradation [80,81]. The adjuvant property of mRNA vaccines can be changed by both the delivery systems and the mRNA itself, which makes using these vaccines difficult for future uses. Therefore, the requirement for better drug delivery mechanisms or vectors continues to impede the wider implementation of mRNA-based treatments [82]. Because vaccines are extremely sensitive to temperature, it is critical to store and transport them within an appropriate temperature range from the point of manufacture to the point of administration, to ensure their effectiveness [83,84]. Vaccines must typically be stored and transported in a cold chain; however, the supply chain for mRNA vaccines may require even lower temperatures. For mRNA vaccines, the requirement for such cold storage still presents a difficulty [85]. Due to the instability of the LNP–mRNA system, mRNA vaccines must be stored at a specific temperature. The poor stability, low translational efficiency, and poor cell targeting of naked mRNA can be addressed by sophisticated delivery devices [86]. The lack of a delivery system in many clinically evaluated mRNA vaccine candidates, however, indicates that mRNA vaccine delivery technologies still require refinement.

## 8. Conclusions

In this new scientific era, mRNA vaccines have become a promising technological platform for vaccine development. The COVID-19 pandemic has advanced the development of mRNA vaccine platforms for the prevention and treatment of a range of infectious diseases. As a result, a new generation of vaccines has gradually gained popularity and access to the general population. To adapt the design of antigen, and even combine various sequences from different variations in response to latest changes in the viral genome, mRNA vaccines may be used.

## 9. Further Directions

Current mRNA vaccines provide adequate safety and protection, but the durability of protection will only be known if further clinical studies are conducted. Therefore, it is necessary to investigate and improve mRNA vaccine stability.

## Figures and Tables

**Table 1:** List of ongoing clinical trials evaluating the role of mRNA vaccines in cancer.

Clinical Trial ID	Study Type	Phase	Population	Groups	Primary Outcomes
NCT05968326	Multicenter randomized	II	Patients (*n* = 260) with resected pancreatic ductal adenocarcinoma	Autogene cevumeran (mRNA) + atezolizumab + folfirinox Folfirinox alone	Disease-free survival
NCT03897881	Randomized	III	Patients after complete resection of high-risk melanoma	mRNA-4157 + pembrolizumab Pembrolizumab alone	Recurrence-free survival (RFS), assessed using radiological imaging
NCT05198752	Open label	I	Patients with advanced malignant solid tumours	SW1115C3 (mRNA)	Dose-limiting toxicity incidence
NCT04382898	Randomized multicenter four-arm	I/II	Patients with high-risk, localized prostate cancer	BNT112 BNT112 + cemiplimab	Dose-limiting toxicity, adverse events, objective response rate
NCT05192460	Single-center, single-arm	Not applicable	Patients with advanced gastric cancer, esophageal cancer, and liver cancer	mRNA + PD-1/L1 mRNA alone	Adverse events, objective response rate

## Data Availability

Not applicable.
